# Filovirus Entry: A Novelty in the Viral Fusion World

**DOI:** 10.3390/v4020258

**Published:** 2012-02-07

**Authors:** Catherine L. Hunt, Nicholas J. Lennemann, Wendy Maury

**Affiliations:** Department of Microbiology, University of Iowa, Iowa City, IA 52242, USA; Email: catherine-l-miller@uiowa.edu (C.L.H); nicholas-lennemann@uiowa.edu (N.J.L)

**Keywords:** ebolavirus, marburgvirus, filovirus, virus entry, virus fusion, endocytosis, TIM-1, NPC1

## Abstract

Ebolavirus (EBOV) and Marburgvirus (MARV) that compose the filovirus family of negative strand RNA viruses infect a broad range of mammalian cells. Recent studies indicate that cellular entry of this family of viruses requires a series of cellular protein interactions and molecular mechanisms, some of which are unique to filoviruses and others are commonly used by all viral glycoproteins. Details of this entry pathway are highlighted here. Virus entry into cells is initiated by the interaction of the viral glycoprotein_1_ subunit (GP_1_) with both adherence factors and one or more receptors on the surface of host cells. On epithelial cells, we recently demonstrated that TIM-1 serves as a receptor for this family of viruses, but the cell surface receptors in other cell types remain unidentified. Upon receptor binding, the virus is internalized into endosomes primarily via macropinocytosis, but perhaps by other mechanisms as well. Within the acidified endosome, the heavily glycosylated GP_1_ is cleaved to a smaller form by the low pH-dependent cellular proteases Cathepsin L and B, exposing residues in the receptor binding site (RBS). Details of the molecular events following cathepsin-dependent trimming of GP_1_ are currently incomplete; however, the processed GP_1_ specifically interacts with endosomal/lysosomal membranes that contain the Niemann Pick C1 (NPC1) protein and expression of NPC1 is required for productive infection, suggesting that GP/NPC1 interactions may be an important late step in the entry process. Additional events such as further GP_1_ processing and/or reducing events may also be required to generate a fusion-ready form of the glycoprotein. Once this has been achieved, sequences in the filovirus GP_2_ subunit mediate viral/cellular membrane fusion via mechanisms similar to those previously described for other enveloped viruses. This multi-step entry pathway highlights the complex and highly orchestrated path of internalization and fusion that appears unique for filoviruses.

## 1. Introduction

Filoviruses (family *Mononegavirales*, genera *Ebolavirus* (EBOV) and *Marburgvirus* (MARV)) are single-stranded, negative-sense RNA viruses that exhibit a unique heterogeneous filamentous structure. Both EBOV and MARV infect a wide variety of mammals and this wide tropism has complicated the identification of cellular proteins required for viral entry. A hemorrhagic fever is caused by these viruses in humans, non-human primates and perhaps other mammals and is associated with high morbidity and mortality during outbreaks. No therapeutic drugs or vaccines are currently available to treat or prevent filoviral infection. Because of this and the high lethality associated with infection, filoviruses are considered Category A Priority Pathogens by NIAID and, in recent years, much research has focused on understanding how these viruses bind to and enter permissive cells.

## 2. Synthesis of Filoviral GPs

The EBOV genome encodes seven genes, including the glycoprotein (GP) gene. The GP gene encodes for two known soluble GP forms (sGP and ssGP) [1,2]. The precise functions of the soluble forms of EBOV GP are unknown, but it is speculated that they are involved in immune evasion, as most of the antibodies during a natural Zaire EBOV (ZEBOV) infection are against sGP [3]. Full-length EBOV GP is not directly encoded by the GP gene, but is produced by a transcriptional editing event that shifts the reading frame of about 20% of the GP transcripts. In contrast, while MARV also encodes seven genes, its GP is directly encoded by the GP gene and soluble forms of MARV GP are not thought to be synthesized from viral transcripts. 

Precursor GP is cleaved by the host enzyme furin in the Golgi apparatus, resulting in the formation of two GP subunits, GP_1_ and GP_2_. GP_1_ contains the receptor binding domain (RBD) and is responsible for interacting with one or more cellular receptors. This interaction is believed to mediate virus entry into the endosomal compartment. GP_2_ contains a fusion loop, heptad repeat regions, the transmembrane domain and a short cytoplasmic tail. While furin processing of the filovirus GP routinely occurs within the Golgi apparatus before the glycoprotein is expressed on the plasma membrane, proteolytic clipping is not required for virion infectivity [4]. The cleaved subunits are linked by a disulfide bond to generate a GP_1,2_ heterodimer that is located on the surface of virions and is approximately 150 kDa in size [5]. 

## 3. Structure of Filovirus GPs

As a class I viral fusion protein, GP_1,2_ exists as a trimer of GP_1_ and GP_2_ heterodimeric subunits and is found in its full-length, heavily glycosylated, pre-fusion form on the surface of newly budded virions. This pre-fusion class I glycoprotein conformation is thought to be a metastable state, that upon the appropriate set of triggers, will convert to its post-fusion, low-energy form [6]. The conformational change from a pre-fusion to post-fusion structure provides the energy to permit the viral and cellular membranes to fuse and thereby release the viral core into the cytoplasm. The events that are required for filovirus/cellular membrane fusion to occur have yet to be completely elucidated, but current studies are unraveling these steps and are highlighted here. 

Several EBOV GP crystal structures have been instrumental in understanding the conformational alterations that are required as the glycoprotein changes from the metastable, pre-fusion state to the low-energy, post-fusion form. Lee *et al.* elucidated the trimeric structure of pre-fusion, mucin domain-deleted EBOV GP_1,2_ ectodomain [7], whereas, two groups independently solved the trimeric GP_2_ six helix bundle structure that is formed during fusion events [8,9].

## 4. GP_1_

**Figure 1:**
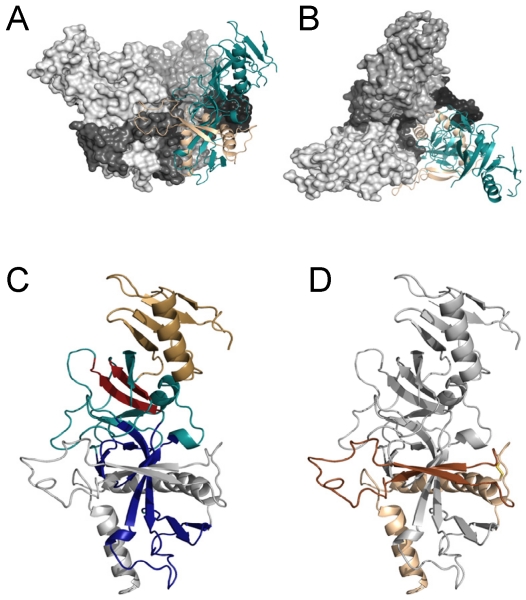
Structure of the pre-fusion EBOV GP. (**A**,**B**) Structure of the trimer. Side view of the EBOV GP trimer is shown in A. Top-down view of the EBOV GP trimer is in B. Two of the three trimers are shown as space filling structures with GP_1_ in lighter grey and GP_2_ as dark grey/black. The third GP_1,2_ heterodimer of the trimer is depicted as a ribbon structure with GP_1_ shown in teal and the GP_2_ subunit shown in tan. (**C**,**D**) Ribbon diagrams of a single heterodimer of GP_1,2_. Domains in GP_1_ are highlighted in C, whereas domains in GP_2_ are highlighted in D. In panel C, the base domain of GP_1_ that interacts with GP_2_ is shown in royal blue, the head domain is shown in teal with the beta-strands and adjacent loop region containing the RBS highlighted in red and the glycan cap is shown in gold. GP_2_ is shown in grey. In panel D, the internal fusion loop (IFL) that is flanked by beta-strands is shown in dark brown and heptad repeat region 1 is shown in tan. The interaction of the IFL with GP_1_ residues from an adjacent subunit is evident in panel A. All EBOV GP graphics (PDB accession number 3CSY) were produced with PyMol.

Mature GP_1_ is composed of three distinct domains: the RBD, the glycan cap and a heavily O-linked glycosylated mucin-like domain. The RBD in mature ZEBOV GP_1_ is located from amino acid ~54 to 201 and composed of a base region and a region that interacts with one or more receptors on the surface of cells (Figure 1) [10]. While RBDs of the different EBOV strains are relatively conserved, only 47% identity between EBOV and MARV RBDs exists. Nonetheless, EBOV and MARV GP pseudovirions compete with each other for filoviral GP_1,2_-dependent entry into permissive cells, indicating that a common receptor or receptors are used by both viruses [10,11].

Amino acids 33 through 69 and three additional short downstream regions interact with GP_2_, serving as the base of the RBD. Several linearly discontinuous regions from amino acids 70 to 190 sit above the base forming a series of beta sheets. Residues both in beta sheets and adjacent loops have been implicated in cell binding, leading to the conclusion that the receptor binding site (RBS) is located in this region of the RBD [12,13]. GP_1_ residues 227 to 313 encode for a “glycan cap” that is extensively N-linked glycosylated and sits distal to the RBD from the surface of the virion. The glycan cap may protect the RBS from antibodies [3]. This glycan cap also interacts with two regions of GP_2_, including the internal fusion loop of GP_2_ that is critical for GP_2_-mediated membrane fusion [7]. The glycan cap/GP_2_ interaction restricts the availability of the fusion peptide, preventing pre-mature fusion events. Finally, filovirus GP_1_ contains a mucin-like domain at the carboxy terminus from amino acids 310 to 511. This region is heavily glycosylated with both N- and O- linked glycans [7]. While the EBOV mucin domain is not required for virus entry [14,15], several roles for this domain have been suggested. Like the glycan cap, it may shield GP RBS residues from immune recognition on free virus [7]. In addition, the mucin domain causes cell rounding, masking of a number of cell surface markers and cytotoxicity that is not observed upon expression of mucin domain-deleted EBOV GP [15,16]. The shielding effect of the bulky mucin domain of the RBD of GP_1_ is thought to also obstruct RBS interactions with adherence factors and receptors since removal of this domain enhances EBOV titers. Similar attempts to delete the MARV mucin domain have proved unsuccessful [11]. 

## 5. GP_2_

Filovirus GP_2_ is similar to other class I viral glycoproteins that mediate fusion events. GP_2_ is composed of a fusion loop found near the amino terminus followed by a N-terminal heptad repeat region, a C-terminal heptad repeat region, a transmembrane region and a short cytoplasmic tail [6,8,9]. In the pre-fusion form on the surface of newly budded virions, the fusion loop and N-terminal heptad region are integral regions of the GP_1_/GP_2_ trimeric structure, forming a platform or base upon which GP_1_ sits (Figure 1). In contrast, the C-terminal heptad repeat region in the pre-fusion form may contain little structure and was deleted in the crystallization studies and it is not thought to contribute to the metastable, pre-fusion form of GP [7]. Not surprisingly, both the fusion loop and N-terminal heptad repeat are conserved (>70% identity) between ZEBOV and the Musoke strain of MARV, but the remaining portions of GP_2_ have limited identity. Site directed mutagenesis studies of EBOV or MARV GP_2_ [14,17,18,19,20,21,22,23] suggest a similar chain of events to those previously reported for other class I viral glycoproteins leads to viral/cell membrane fusion [6,24,25]. These events will be discussed in detail below. 

## 6. The Role of Cell Surface Proteins in Filovirus GP-Dependent Entry

While progress is being made to identify cell surface proteins that enhance filovirus transduction/infection, the advancement of this area of research has been slow. In part this is due to the broad tropism of filoviruses for a variety of different cell types as well as the ability of these viruses to infect cells from a wide range of species [26,27,28,29,30,31,32,33,34,35,36,37]. One classical approach to identifying virus receptors that has been used is the introduction of a cDNA library from a permissive cell into a cell that is not permissive for the virus [38,39,40]. However, for reasons that are not entirely clear, this type of study has not been successful in identifying cell surface proteins that directly interact with EBOV GP to mediate virus entry [41,42]. Instead, another screening approach that correlated gene expression in a large panel of human cells with EBOV GP-dependent transduction proved more productive and allowed us to identify a surface receptor for these viruses [43]. The cell surface protein we identified, TIM-1, as well as other cell surface proteins that enhance filovirus infectivity are discussed in detail below. 

### 6.1. C-Type Lectins

C-type lectin family members L-SIGN, DC-SIGN and hMGL have been shown to enhance filovirus entry [19,44,45,46,47,48]. Studies have demonstrated that both the mucin domain and the glycan cap of GP_1_ interact with C-type lectins [47,49]. However, as both of these regions can be deleted from EBOV GP_1_ without loss of viral transduction efficiency [16,50,51,52], it is likely that C-type lectins increase filovirus attachment to cells rather than serving as cellular receptors that mediate internalization of the virus into endosomes [53]. A similar adherence function for C-type lectins has been identified for other enveloped viruses such as HIV [54].

### 6.2. β1 Integrins

Early studies found that surface expression of some integrins was down regulated upon transfection of full-length EBOV GP [15,55,56]. In addition, EBOV GP-mediated pseudovirion entry is reduced by either antibodies targeting β1 integrins or a soluble form of β1 integrins [56]. These characteristics suggested that one or more integrin subunits might serve as receptors for filoviruses. However, no direct interaction between any portion of EBOV GP and a member of the β1 integrin family has been identified. More recent studies implicate β1 integrin in stimulation of endosomal protease events that are required for productive EBOV transduction, thus reducing the likelihood of β1 integrins serving as bona fide filoviral receptors [57]. 

### 6.3. Tyro3 (TAM) Family Tyrosine Kinase Receptors

The TAM family member Axl was first implicated in filovirus entry through a cDNA screen that introduced Vero E6 cell cDNA into poorly permissive Jurkat T cells [41]. Axl is a tyrosine kinase receptor that is found on the plasma membrane in a variety of different cell types and enhances cell migration, division and viability upon activation [58]. Shimojima *et al.* demonstrated that anti-Axl antibodies blocked EBOV transduction of some cells, whereas these antibodies had no effect on transduction of other cells [41]. Mapping studies indicated that amino acid residues in both the ectodomain and the cytoplasmic tail of Axl were required for filovirus entry enhancement [59]. Subsequently, a screen performed in our laboratory also identified Axl as being important in EBOV GP-dependent entry [60]. Through the use of multiple biochemical inhibitors, siRNA and anti-Axl antibodies, we defined a role for Axl in EBOV uptake [61], demonstrating that Axl expression enhances macropinocytosis in some cells. As macropinocytosis is a principal uptake mechanism of filoviruses [62,63], increased Axl surface expression leads to greater virus internalization. However, Axl does not appear to interact directly with EBOV GP to promote viral internalization and therefore is unlikely to serve as a filoviral receptor [60]. 

### 6.4. TIM-1

Our lab performed a comparative genomic analysis (CGA) screen [64] to identify cellular genes whose expression highly correlated with EBOV pseudovirion transduction [43]. This screen showed a positive correlation between EBOV transduction and expression of a series of cellular proteins that were previously appreciated to enhance EBOV transduction (C-type lectins, integrins and Axl). Interestingly, expression of the T-cell immunoglobulin mucin domain-1 (TIM-1) gene proved to be one of the strongest positive correlations. Subsequent studies demonstrated that expression of TIM-1 in poorly permissive cells enhanced EBOV entry and loss of surface-expressed TIM-1 in highly permissive cells abrogated filovirus infection/transduction. Furthermore, TIM-1 and the mucin domain-deleted EBOV GP interacted, and removal of the glycan cap enhanced the specificity of GP interaction with TIM-1-expressing cells [43]. In total, these findings have led us to propose TIM-1 as a cell surface receptor for filoviruses. As epithelial cells are the only relevant cell type that expresses TIM-1, it is likely that other as of yet unidentified surface receptors will also prove to be important in mediating filovirus entry. 

## 7. Mechanism(s) of Filoviral GP-Mediated Entry into Permissive Cells

The cellular uptake pathways mediating filovirus entry remain controversial despite numerous studies. The three most common and well studied endocytic pathways—caveolin-dependent endocytosis, clathrin-dependent endocytosis and macropinocytosis—have all been implicated in filovirus entry. Early studies reported that the caveolae vesicular system and/or lipid raft domains were important for EBOV GP-mediated entry [65,66,67]. However, it was demonstrated that overexpression of Caveolin 1 in the poorly permissive lymphocytic cell line CEM did not enhance levels of EBOV GP-dependent transduction, suggesting that caveolae may not play a role in filovirus entry [36]. Since that time several groups have also implicated clathrin-dependent entry mechanisms in filovirus entry [68,69,70,71]. In parallel, other groups using virus-like particles (VLPs) and/or infectious filovirions identified macropinocytosis as a main and perhaps sole mechanism of filovirus uptake [62,63]. Several groups have found that EBOV GP-dependent entry can occur through a variety of different uptake mechanisms including caveolae, clathrin-coated pits and through actin- and microtubule-dependent pathways such as macropinocytosis [61,70,71] within the same cell population. 

A number of these uptake studies that identified caveolin and/or clathrin as important in entry used EBOV GP pseudotyped lentiviruses or vesicular stomatitis virus (VSV) as the cargo and an argument has been made that these smaller viral particles do not accurately represent the size constraints that may limit the uptake options of a filovirion that can be greater than 10 µM in length [72]. While a role for caveolin- and clathrin-dependent pathways in infectious filoviral entry remains to be demonstrated, it should be noted that several large, intracellular bacteria or parasites have been found to use either caveolin- or clathrin-dependent pathways, suggesting that the size of these vesicles can be expanded to meet the size of the cargo [73,74,75]. In addition, it is likely that the mechanism of EBOV uptake may be cell-dependent as both Green African Monkey cells (Vero) [62,63] and human neuroblastoma cells (SNB-19) [61] both primarily use macropinocytosis for EBOV uptake. However, the signaling pathways that are required for virus entry is cell-specific since Vero cells require activation of the PI3K/Akt pathway, whereas SNB-19 cells require PLC activation [61]. 

## 8. Proteolytic Processing of EBOV GP_1,2_ into a Fusion-Active Form

Unlike some other class I viral glycoproteins, no evidence exists that EBOV GP_1_ undergoes conformational changes upon binding to a cell surface receptor. Presumably receptor/virus interactions lead directly to virus internalization into endosomes; however, this has not been demonstrated directly to date. Characterization of the endosomal vesicles mediating EBOV uptake is limited. Saaed *et al.* have demonstrated that the Early Endosome Antigen 1 (EEA1) colocalizes with EBOV virus particles in endosomes early during infection of Vero cells [62], whereas Nanbo *et al.* have shown association of EBOV particles lacking VP30 associated with the sorting nexin 5 (SNX5) within 10 minutes of transduction [63]. Early and late endosomal proteins, Rab5 and Rab 7, respectively, have also been shown to be required for productive infection [62,63]. Several GTPases previously implicated in endocytosis, RhoB, Rac1 and CDC42, are also important for EBOV GP-dependent transduction, providing additional insights into trafficking pathways used by these viruses [63,76]. 

As the vesicles traffic into the cell, they become acidified. For some viral fusion proteins, the combination of receptor engagement and endosomal acidity is sufficient for conformational changes that lead to viral/cellular membrane fusion [77,78,79]. However, that is not the case for filoviruses. Instead, multiple low pH-dependent endosomal and lysosomal proteolytic proteins are involved in EBOV GP_1_ processing, priming a multi-step process that ultimately results in a small EBOV GP_1,2_ trimer that serves as the fusion-ready form of the glycoprotein. It is believed that this processed, fusion-ready form is then triggered by additional events to a conformationally stable state, resulting in fusion.

As endosomal vesicles mature into late endosomes and the vesicular pH drops, activation of endosomal cysteine proteases Cathepsin L and B occurs. These cathepsins sequentially process EBOV GP_1_ into smaller forms [50,51]. Cathepsin L proteolysis first removes a substantial portion of EBOV GP_1_, generating an approximate 50 kDa GP_1,2_ species that lacks both the glycan cap and mucin domain of GP_1_ [50,51]. Subsequently, GP_1_ is further trimmed by both Cathepsin L and B to generate a much smaller form of GP_1_. The exact size of this smaller GP_1_ remains controversial, but is between 17 and 19 kDa [50,51]. Irrespective of the exact size of the processed form, prevention of endosomal acidification or inhibition of Cathepsin B activity abolishes EBOV infectivity [50,51,52,70]. Interestingly, this processing may be insufficient for productive EBOV infection as studies have demonstrated that the smaller 17–19 kDa Cathepsin L and B-cleaved form cannot infect cells entirely lacking cathepsins [80]. Thus, it has been proposed that additional cathepsin-dependent GP_1_ processing is required to generate the fusion-ready form of the glycoprotein [80]. The size and composition of this smaller form is not known. In contrast to these studies, a recent study demonstrated that a thermolysin-trimmed GP_1_ that is believed to generate a GP_1_ that is similar to the Cathepsin L and B-cleaved form can be triggered to bind to liposomes at elevated temperatures under low pH and mildly reducing conditions [81]. This new study suggests that at least under these conditions this GP conformation is a fusion-ready form.

Given the apparent importance of cathepsin cleavage for the generation of a fusion-ready form of the filovirus glycoprotein, it is surprising that studies have demonstrated that Cathepsin L and B cleavage events can be sidestepped by the virus [80,82]. Martinez *et al.* have shown that in monocyte-derived dendritic cells, Cathepsin B is required for EBOV infection, but not Cathepsin L [82]. These observations were confirmed in Vero cells in a recent study by Wong *et al.* [80]. This group also extended the finding by selecting an infectious, recombinant VSV encoding EBOV GP over several passages to become resistant to the Cathepsin B inhibitor CA074 [80]. Six different mutants were identified that conferred Cathepsin B independence. Many of these mutations sit at the interface of GP_1_ and GP_2_ and the selected mutations were thought to alter the GP structure such that enhanced proteolysis by one or more currently undefined cysteine proteases was possible. Thus, while Cathepsin B and L independence can be achieved by filoviruses, processing by one or more additional cysteine proteases is still required for production of the fusion-ready form. 

## 9. NPC1

Most recently, two groups independently identified a novel host protein essential for EBOV infection. Cote *et al.* screened a library of small chemical molecules to find those that inhibited EBOV GP pseudovirion entry into Vero cells [83], whereas Carette *et al.* utilized a genome-wide screen in mutagenized haploid human cells to look for those cells resistant to EBOV GP-dependent entry [84]. Each group was able to deduce that disruption of one endosomal/lysosomal membrane protein, Niemann-Pick C1 (NPC1), could significantly reduce EBOV entry into a variety of cell populations.

NPC1 is primarily a membrane bound, late endosomal/lysosomal protein that is critical for cholesterol absorption and homeostasis. Those individuals lacking a functional NPC1 exhibit an abnormally high accumulation of cholesterol in the lysosomes of their cells, leading to altered protein and lipid trafficking with most cases resulting in fatality by 18 months of age [85]. Cells where NPC1 function has been biochemically disrupted or cells lacking NPC1 showed a resistance to EBOV infection [83,84]. Clearance of cholesterol from NPC1 null cells by cultivation in lipoprotein-depleted media did not rescue EBOV infection, indicating that the NPC1 protein *itself*, and not aberrant cholesterol transport, was important for EBOV entry [84]. Cote *et al.* also showed that the expression of NPC1 and not its cholesterol transport activity were critical for EBOV entry [83]. 

As the NPC1 protein is primarily located on the endosomal and lysosomal membranes, NPC1 has been proposed to serve as an entry factor downstream of EBOV GP engagement of attachment factors/receptor(s) at the cell surface. Consistent with a vesicular role for NPC1, Cote *et al.* showed that a soluble form of thermolysin-cleaved EBOV GP, but not uncleaved GP containing the glycan cap, bound to lysosomal membranes of NPC1-transfected CHO cells [83]. Thus, at least in this over expression system, proteolytically-processed EBOV GP appeared to interact with NPC1-containing membranes, suggesting that these interactions may be important for filovirus entry events that occur in late endosomes and/or in lysosomes. EBOV GP-mediated attachment and entry into early endosomes was unaffected in NPC1-defective cells; however, electron micrographs of NPC1 null cells infected with EBOV GP pseudotyped virus show the accumulation of perinuclear vesicles laden with EBOV GP pseudovirions that were positive for the lysosomal marker LAMP1 [84]. Therefore, Carette *et al.* have proposed that NCP1 is crucial for viral membrane fusion and escape from the lysosomal vesicle [84]. At present, the precise role of NPC1 during the EBOV entry process remains to be fully elucidated. In addition, investigations into the precise location of filovirus fusion events within endosomal compartments will provide important insights into these events.

Interestingly, an inhibitor of NPC1, U18666A, has recently been shown to block entry of several pathogens, including the flavivirus Dengue [86]. This inhibitor also inhibited entry of hepatitis C virus [87] as well as prions [88], suggesting that this cholesterol transporter may be critical for passage of a number of viruses, and perhaps other pathogens, through endosomes. Most recently, a more definitive role for NPC1 in hepatitis C virus entry has been determined through the use of additional biochemical agents [89]. Future studies exploring lipid accumulation and changes in lipid composition within the endosomal pathway could significantly enhance the understanding of the novel role of NPC1 specifically in filoviral entry and more generally in endosomal trafficking of a number of enveloped viruses. 

## 10. Characterized Filovirus Fusion Events

As described above, the GP_1_ portion of EBOV GP_1,2_ allows delivery of the filovirion to an endosome where conditions become progressively more favorable for generating the fusion-ready form of GP_1,2_. Trimming of GP_1,2_ by host cathepsins (or artificially by thermolysin) enhances interaction of GP with TIM-1 [43] and permits NPC1 binding [83]. Whether EBOV GP interaction, with either of these molecules, directly mediates GP_2_ fusion events remains to be determined.

Filovirus GP_2_ contains an N-terminal internal fusion loop of 45 residues defined by a disulfide linkage between cysteines 511 and 556 [3,7]. A core hydrophobic sequence of 16 amino acids within this loop is thought to insert into host endosomal membranes, initiating membrane fusion events. Within the intact GP heterodimer, the hydrophobic, internal fusion loop is flanked by antiparallel β-strands composed of the most N-terminal portion of the internal fusion loop and the N-terminal region of first heptad repeat region. The fusion loop is restrained by GP_1 _residues from a neighboring subunit, preventing premature fusion events [3,7,90]. Cathepsin-dependent processing alone is insufficient to trigger insertion of the fusion loop into liposomes [81,91]. 

When the EBOV internal fusion loop interacts with liposomal membrane mimetics, lipid mixing is promoted with a parallel structural change in the loop [90]. In a neutral pH, lipid environment, the antiparallel β-strands that flank the fusion loop lose their structure, generating a more alpha helical content with a flattened extended loop structure. Under low pH conditions (<pH 5.5) in the presence of lipids, this flattened loop structure broadens out, forming a more hook-like structure [90]. It has been previously shown that specific proline residues contained within the central portion of the fusion loop facilitate this lipid membrane interaction [21,23,92,93]. The insertion of GP_2_ into host membranes causes an extension of the GP_2_ trimers into an energetically unfavorable state. This also causes the two heptad repeat regions (HRs) within GP_2_ to be fully exposed to the physiological conditions within the acidified host endosome, which may aid in further triggering of GP_2_ to promote final collapse of the two HRs [81]. 

The N-terminal GP_2_ heptad repeat region (HR1) is a highly ordered, alpha-helical structure that serves as a platform or base for GP_1_ and contains residues that are necessary for interactions with other GP_2_ HR1s to form the trimeric structure of the pre-fusion GP [3,7]. In the pre-fusion form, HR1 does not interact with the carboxy terminal heptad repeat region (HR2). Within the intact, pre-fusion trimer, HR2 appears to be disordered and was not included in the crystallized structure [7]. 

Following insertion of EBOV GP_2_ fusion loop into the host membrane, the EBOV GP_2_ trimeric heptad repeats collapse and form a six-helix bundle containing three HR1 and HR2 domains. The collapse into a coiled coil structure draws the two membranes into proximity allowing partial fusion (hemifusion) of the viral membrane and the host endosomal membrane [8,9]. Hemifusion eventually leads to complete fusion of the viral and host endosomal membranes, and an opening through which the viral RNA and its associated proteins can be released into the host cell cytoplasm, where the viral life cycle continues.

## 11. Potential Therapeutics against Filovirus Entry

**Figure 2:**
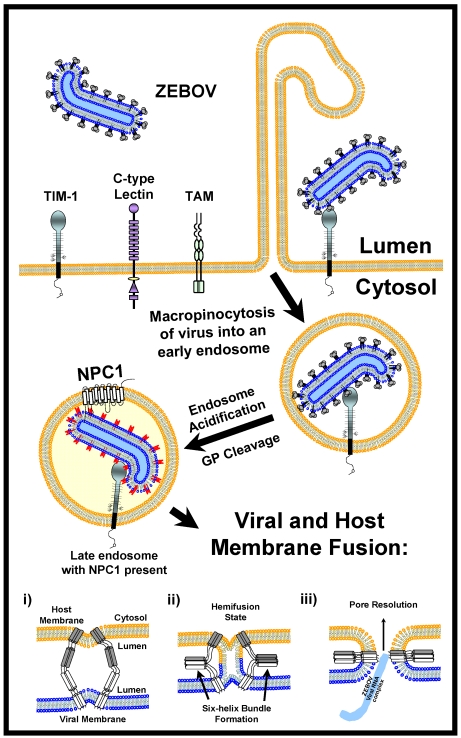
Model for filoviral entry. Trimers of filoviral GPs on virions interact with both attachment factors (C-type lectins) and receptors (TIM-1) on the surface of permissive cells. Attachment factors are likely to concentrate virions on cells before receptor engagement and virion internalization by macropinocytosis. Macropinocytosis is enhanced by tyrosine kinase receptors such as TAM family members. Following endosomal acidification, Cathepsins L and B trim the EBOV GP to a smaller form that needs at least one as yet undetermined factor to elicit GP fusion with host endosomal membranes. This smaller form of GP is able to interact with both TIM-1 and the endosomal portion of the NPC1 protein; however, whether GP and TIM-1 interact within endosomes is not known. The energetically unfavorable insertion of the EBOV GP_2_ fusion loop into host endosomal membranes (**i**) is followed by the energetically favorable collapse of EBOV GP into a six-helix bundle (**ii**) allowing for lipid mixing and hemifusion of host and viral membrane lipids (ii). Finally, the hemifused host and viral membranes resolve and a complete pore is formed (**iii**) through which the viral genomic complex passes into the cytoplasm, allowing the viral replication cycle to continue.

This complex set of entry events summarized in Figure 2 provides numerous potential avenues for the development of antiviral therapies against filovirus infection. These possibilities include: (1) interfering with adherence to permissive cells by blocking C-type lectin (or other adherence factor) interactions with the filoviral GP; (2) blocking binding of GP to TIM-1 and/or other cellular receptors that are identified in the future; (3) preventing virus uptake by blocking macropinocytosis; (4) interfering with cathepsin activity; (5) inhibiting availability of NPC1 within the lysosomal compartment and (6) blocking HR1/HR2 (coiled coil) interactions. It is likely that some of these steps will be more amendable than others to the development of antivirals that have minimal or no cytotoxicity. Regardless, elucidation of these entry events provides clear targets for the development of drugs that may prevent both filovirus infection and disease.
